# Karyotypes of *Chironomus* Meigen (Diptera: Chironomidae) species from Africa

**DOI:** 10.3897/compcytogen.v5i1.975

**Published:** 2011-05-05

**Authors:** Wolfgang F. Wülker, I.I. Kiknadze, A.G. Istomina

**Affiliations:** 1 Zur Bitzenmatte 9, D79249 Merzhausen, Germany; 2 Institute of Cytology and Genetics, Russian Academy of Sciences, Siberian Branch, Novosibirsk, 630090, Lavrentiev pr. 10, Russia

**Keywords:** *Chironomus*, karyotype, banding sequences, chromosomal polymorphism, chromosomal evolution

## Abstract

The karyotypes of six African *Chironomus* species (*Chironomus alluaudi* Kieffer, 1913, *Chironomus transvaalensis* Kieffer, 1923, *Chironomus* sp. Nakuru, *Chironomus formosipennis* Kieffer, 1908, *Chironomus prope pulcher* Wiedemann, 1830, *Chironomus* sp. Kisumu) were investigated; four of these karyotypes were described for the first time (*Chironomus* sp. Nakuru, *Chironomus formosipennis*, *Chironomus prope pulcher*, *Chironomus* sp. Kisumu). Of the six *Chironomus* karyotypes, three had “pseudothummi” cytocomplex chromosome arms combinations AE CD BF G (*Chironomus alluaudi*, *Chironomus transvaalensis*, *Chironomus* sp. Nakuru), two had “thummi”cytocomplex arms combinations AB CD EF G (*Chironomus formosipennis*, *Chironomus prope pulcher*), and one had “parathummi”armcombinations AC BF DE G (*Chironomus* sp. Kisumu). Thus, three of the ten main cytocomplexes known were detected in Africa. Detailed photomaps of all chromosome arms, with the exception of arms B and G, were prepared for the karyotypes of *Chironomus alluaudi*, *Chironomus transvaalensis*, *Chironomus* sp. Nakuru, *Chironomus* prope *pulcher;* the karyotypes of *Chironomus formosipennis*, *Chironomus* sp. Kisumucould only be fragmentarily mapped.

Endemic African banding sequences were characteristic for most of the chromosomal arms in all species studied. However, basic sequences, which can be present in different *Chironomus* species on different continents (Wülker, 1980; Kiknadze et al. 2008), were also detected also in several African species (*Chironomus alluaudi*, *Chironomus* sp. Nakuru, and *Chironomus formosipennis*). The banding sequences of African species studied allow discussion of the derivation of modern banding patterns from hypothetical species, living before separation of cytocomplexes and continents.

## Introduction

As shown by cytogenetic analysis of chromosomal evolution, the divergence of animal karyotypes during speciation was mainly mediated by para- and pericentric inversions, altering the gene orders in linkage groups (Dobzhansky 1970, White 1977, King 1993, Zdobnov et al. 2002). The other types of chromosomal rearrangements (translocations, fusions, duplications) play an additional role in rearrangements of the linear structure of genome. Alteration of the gene orders in chromosomes during evolution can be visualized in Diptera, which possess polytene chromosomes with distinct banding sequences. The bands of polytene chromosomes, which form species-specific banding sequences, are considered as genetic markers to analyze divergence patterns of the linear genome structure during evolution. The use of the number of chromosomal breakpoints as a divergence measure provided establishment of phylogenetic relationships between species (Kiknadze et al. 2008). Species of the genus *Chironomus* have four giant chromosomes with seven chromosome arms (A-G). Based on the different combination of the arms, caused by whole-arm translocations, the *Chironomus* species are grouped into several cytocomplexes (Keyl 1962, Wülker 1980). Cytocomplex is not a taxonomic term. It includes the species with definite chromosome arms combinations, but not similar morphologically. Comparison of banding sequences between species from different cytocomplexes have shown that karyotypes can include species-specific sequences and so called basic sequences, common to more than one cytocomplex and in more than one continent. Such basic sequences were probably present before the separation of species and cytocomplexes (Keyl 1962, Wülker 1980).

By global analysis of banding sequences in Eurasia, North and South America, Australia, we have traced banding sequence changes during *Chironomus* species divergence and continent dispersal (Martin et al. 1974, Wülker 1980, Wülker et al. 1989, Kiknadze at al. 2003, 2008). It was shown that in Eurasia, North America, and Australia, banding sequence pools of many species were represented mainly by endemic continent-specific sequences. However, basic sequences, common for different continents were also found in karyotypes of some species in addition to the endemic sequences. Such basic sequences were noted also in two African *Chironomus* species (*Chironomus alluaudi*, and *Chironomus* sp. Nakuru) (Martin 1979, Wülker 1980). It was of interest to study how often such basic sequences can be found among African species. However, the data on *Chironomus* karyotypes in Africa are very scanty despite there being much information on the morphology of African chironomids. Wülker (1980) has presented photographs of seven chromosome arms of *Chironomus alluaudi*; Martin (1979) has quoted the arm F banding sequence of *Chironomus transvaalensis*; Wülker et al. (1989) included the banding sequences of *Chironomus transvaalensis* arms A, and F in their list of *Chironomus* sequences, and have shown the position of *Chironomus alluaudi* and *Chironomus transvaalensis* on the phylogenetic tree.

This paper contains full descriptions of the karyotypes of six African *Chironomus* species (*Chironomus alluaudi*, *Chironomus transvaalensis*, *Chironomus* sp. Nakuru, *Chironomus formosipennis*, *Chironomus* prope *pulcher*, *Chironomus* sp. Kisumu). Among them four karyotypes are described for the first time (*Chironomus* sp. Nakuru, *Chironomus formosipennis*, *Chironomus* prope *pulcher*, *Chironomus* sp. Kisumu). Detailed photomaps of arms A, C, D, E, and F are presented for *Chironomus alluaudi*, *Chironomus transvaalensis*, and *Chironomus* sp. Nakuru. The chromosome arms could be mapped only partly for *Chironomus formosipennis*, *Chironomus* prope *pulcher* and *Chironomus* sp. Kisumu.

The presence of further basic banding sequences in the karyotypes of African *Chironomus* species was discovered, along with endemic continent-specific (Ethiopian region) sequences.

Evolutionary divergence of “thummi“ and “pseudothummi” cytocomplexes is discussed.

## Material and methods

Forth instar larvae of African *Chironomus* species were used for karyotype study. 35 years ago, one of us (W.W.) had the opportunity to visit Kenya (22.12.1975–16.01.1976). From a base at the house of relatives in Nairobi, he went with family (wife and 3 sons) to collect chironomids to the west to Lake Nakuru and Lake Victoria, to the north to Abrader Mount Ca. 3000 m above N.N., and to the southeast to Tsavo National park, Mombasa and vicinity. Other material was contributed by colleagues: Mount Elgon and Lake Naivasha (Peter N. Cox), Mount Kenya, near 4350 m (scientific excursion of University Erlangen, Germany, under Prof. Dr. Rüppell), Zigi River, Tanzania (Dr. J. Grunewald). The list of collection sites of *Chironomus* larvae is presented in Table 1. We have not identified species *Chironomus* sp. Nakuru and *Chironomus* sp. Kisumu, but the study of the banding sequences of their karyotypes was very important for purpose of our paper.

**Table 1.** Collection sites and number of specimens of African *Chironomus* species.

Species	Collection sites	Collection date	Collector	Number of specimens
*Chironomus alluaudi*	Kenya: drinking troughs brooks and pools at Endebess/Mt. Elgon, mountain lakes W of Nakuru, Aberdare mountains up to 3300 m, ponds at MtKenya 4350 m, near Limuru (north of Nairobi), Athi-river south of Nairobi, Amboseli-park	29.12.75 03.01.76 12.01.76 13.01.76 10.01.76	P. N. Cox, W.Wülker. G. Rüppell W. d’Oleire-Oltmanns,H.Koehler W. Wülker	120
*Chironomus transvaalensis*	Kenya: pool east Lake Victoria, north and south Athi-river near Nairobi, west of Mombasa, Tansania: Kikuwi-river	27.12.75 13.01.76	W. Wülker; J. Grunewald	115
*Chironomus* sp. Nakuru	Kenya: brook south east Lake Nakuru	26.12.75	W. Wülker	9
*Chironomus formosipennis*	Kenya: Lake Naivasha, Tansania: Zigi-river, running waters		P. N. Cox, J. Grunewald	15
*Chironomus prope pulcher*	Kenya: two pools in short distance, River Athi south of Nairobi	13.01.76	W. Wülker	6
*Chironomus* sp. Kisumu	Kenya: flat pools 10 and 22 km east Lake Victoria, together with *Chironomus transvaalensis*, brook in Amboseli-park (Kilaguni)	27.12.75 10.01.76	W. Wülker	7

Larvae were fixed in ethanol-glacial acetic acid (3:1). The technique of chromosome preparation was as usual (Keyl 1962). The identification of chromosome banding sequences follows by Keyl (1962) for arms A, E, and F, and by Dévai et al. (1989) for arms C and D.

To trace the relationship of African *Chironomus* banding sequences with sequences from other continents, we compared them with known basic sequences; if basic sequences for some of species were unknown, we compared them with *Chironomus piger* standard (ST).

We have pointed to previous literature on morphological characteristics of species studied at the beginning of each species description. Most part of the material (larvae, pupa, adults and karyotype slides) is now deposited in Zoologische Staatssammlungen in Münich (Germany).

Equipment of the Center of Microscopy Analysis of Biological Objects of SB RAS in the Institute of Cytology and Genetics (Novosibirsk) was used in accomplishment of this work: microscope “Axiokop” 2 Plus, CCD camera AxioCam HRc, software package AxioVision 4 (Zeiss, Germany).

## Results

### Chironomus alluaudi

Kieffer, 1913

http://species-id.net/wiki/Chironomus_alluaudi

#### Previous reports:

Kieffer 1913, imago.

Freeman 1957, imago.

Wülker 1980, photo of arms A-G

Wülker et al. 1989, phylogenetic position

Kiknadze et al. 2004, list of banding sequences of arms A, C, D, E, and F

#### Karyotype

(Fig. 1a). Haploid number n=4, arm combination AE CD BF G (“pseudothummi” cytocomplex), centromere bands not heterochromatinized, nucleolus in arm G (terminal), at least 3 Balbiani Rings (BRs) on arm G, inversion polymorphism in arms C and G.

**Figure 1a.**
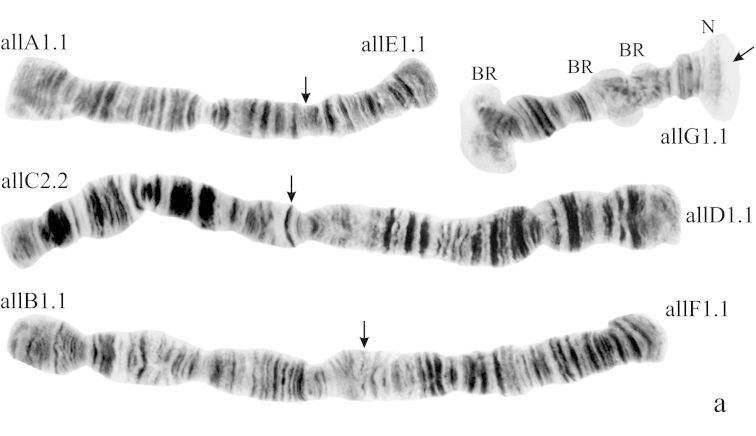
Karyotype of *Chironomus alluaudi*. In this and all other Figures: **allA1.1**, **allE1.1** etc. – symbols of arm and homozygous genotypic combinations **N** – nucleolus **BR** – Balbiani ring, arrows show centromeric bands, brackets near chromosome arms show inversions.

Banding sequences (Fig. 1b-g)

**Figure 1b-g.**
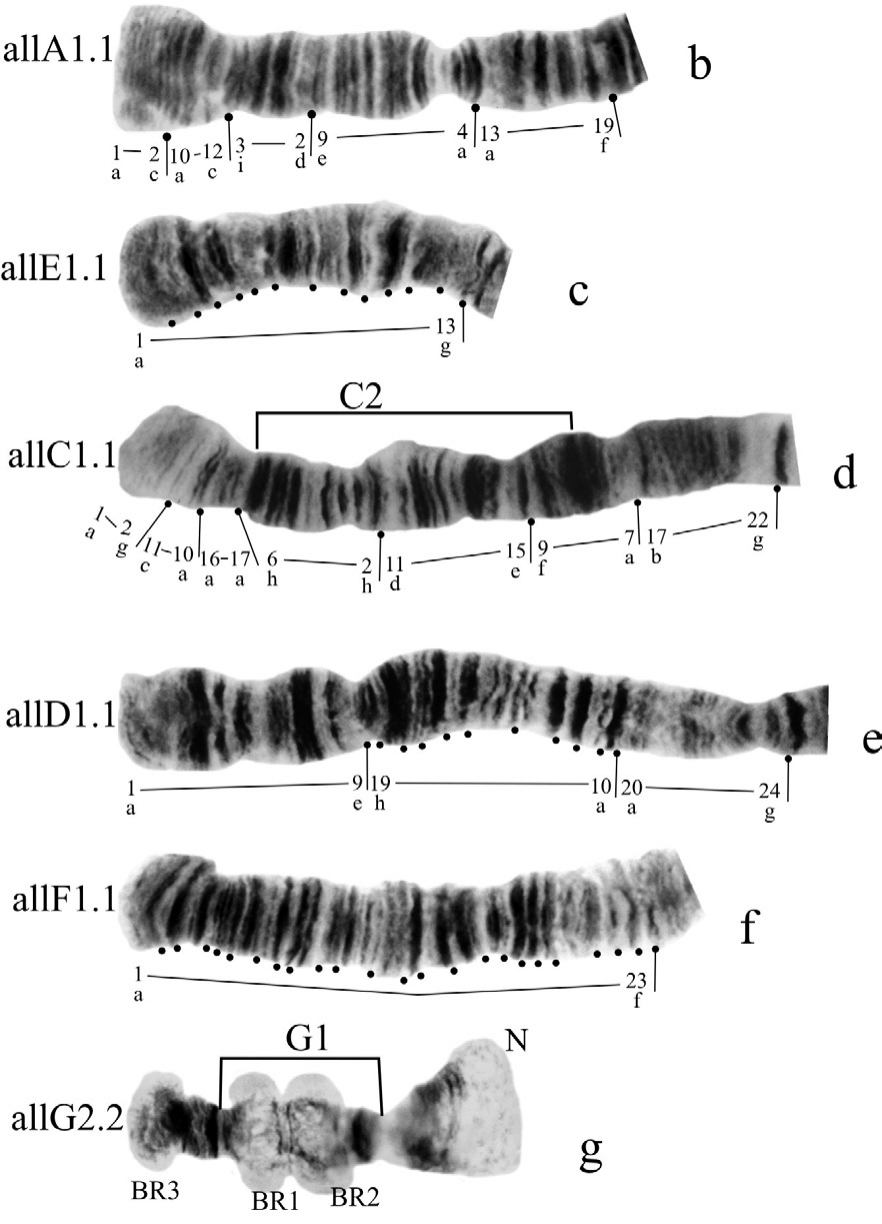
Homozygous banding sequences of *Chironomus alluaudi* in arms A, E, C, D, F and G. The designations are the same as in Fig. 1.

**Arm A** (Fig. 1b) has the sequence all A1 identical with the main sequence of arm A found in many *Chironomus* species (*Chironomus holomelas* Keyl,1961, *Chironomus melanescens* Keyl, 1961, etc.) and it is considered a cosmopolitan basic sequence (holA1).

**Figure:**
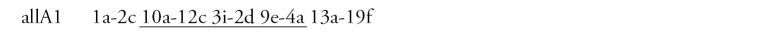


**Arm E** (Fig. 1c, 7b) has the sequence allE1 identical with *Chironomus piger* ST (cosmopolitan basic sequence).

**Figure 2a.**
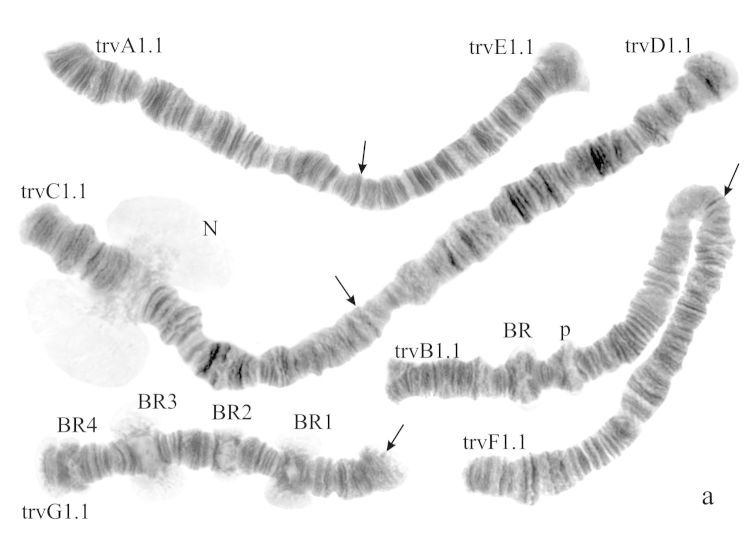
Karyotype of *Chironomus transvaalensis*. **p** – puff and the designations are the same as in Fig. 1

**Figure:**
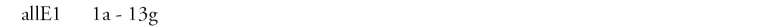


**Arm C** (Fig. 1d) has two sequences, allC1 and allC2, differing by a simple inversion. The sequence allC1 differs greatly from the basic sequence in arm C; therefore we have compared it with *Chironomus piger* ST: differing by seven inversion steps from pigST:

**Figure:**
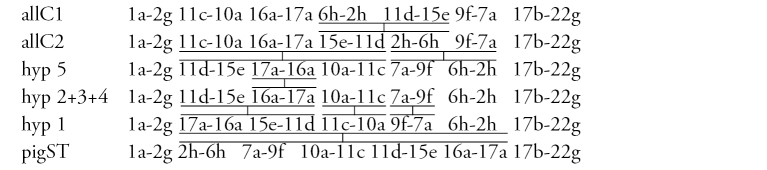


**Arm D** (Fig. 1e) has single sequence allD1 differing by one inversion step from pigST:

**Figure:**
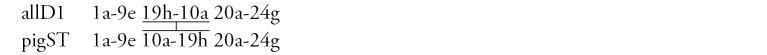


**Arm B** (Fig. 1a) not mapped, monomorphic. The common BR is not developed.

**Arm F** (Fig. 1f) has the sequence allF1, identical with pigST (cosmopolitan basic sequence).

**Figure:**
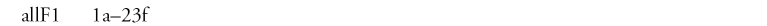


**Arm G** (Figs 1a, g) not mapped, has two sequences allG1 and allG2 differing by one simple inversion in the central part of arm G, including two of the Balbiani rings.

In total, the banding sequence pool of *Chironomus alluaudi* contains 9 sequences. Six of them endemic for Africa (Ethiopian sequences), three of them (allA1, allE1, allF1) belong to the category of cosmopolitan basic sequences. *Chironomus alluaudi* can be considered asa *Chironomus* species with a primitive karyotype (Wülker, 1980, 2010).

#### Larva:

“thummi-type” (no tubuli laterales) on abdominal segment VII). Mentum with high lateral tooth, median tooth as in other *Chironomus* species, pectin epipharyngis about 11 teeth, antenna black with 4 segments, paralabial plates about 40 striae.

#### Distribution:

different places in Africa (Freeman 1957), Kenya (leg. Wülker, Jan. 1976). Dunking troughs brooks and pools at Endebess/Mt Elgon (N. Cox leg.); mountain lakes W of Nakuru, Aberdare mountains up to 3300m, ponds Mt. Kenya 4350m (Oltmanns leg.) near Limuru (north of Nairobi), Athi river south of Nairobi, Amboseli-park (Wülker leg.)

### Chironomus transvaalensis

Kieffer, 1923

http://species-id.net/wiki/Chironomus_transvaalensis

#### Previous reports:

Kieffer 1923, imago.

Mc Lachlan 1969, 1971: larva and pupa.

Freeman 1957, imago.

Martin 1979, banding sequence of chromosome arm F.

Wülker et al. 1989, banding sequences of arms A, E, and F, phylogenetic position of species.

#### Karyotype

(Fig. 2a). Haploid number n=4, arm combination AE CD BF G (“pseudothummi” cytocomplex), centromeric bands not heterochromatinized, nucleolus in arm C, inversion polymorphism in arms C and G.

Banding sequences (Fig. 2b-f).

**Figure 2b-f.**
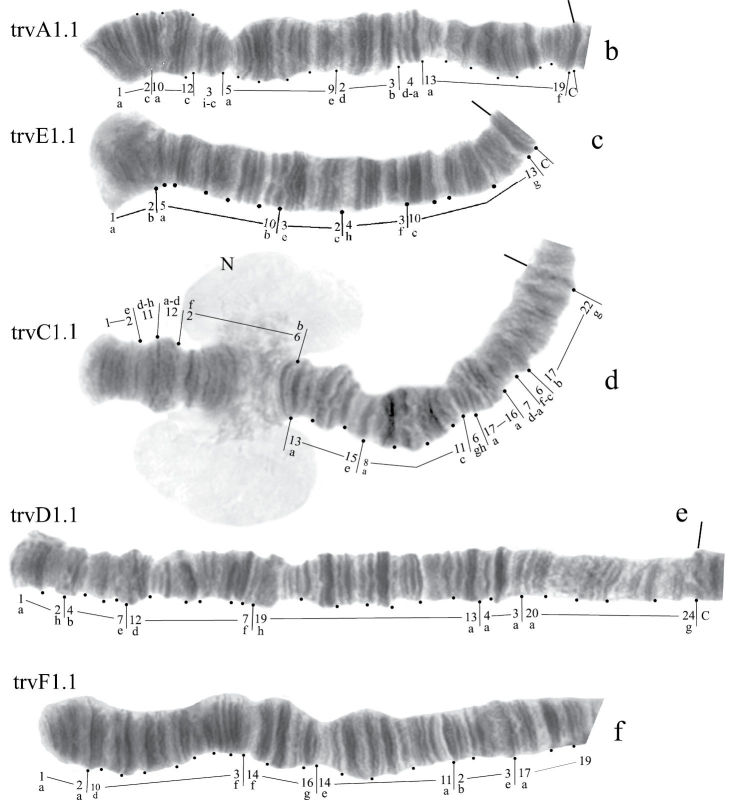
Homozygous banding sequences of *Chironomus transvaalensis* in arms A, E, C, D and F.

**Arm A** (Fig. 2b) has the sequence trvA1, differing by only one inversion step from the basic sequence holA1.

**Figure:**
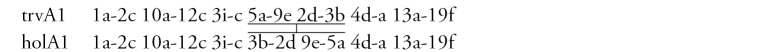


**Arm E** (Fig. 2c) has the banding sequence trvE1, differing only by one step from basic sequence aciE1 (*Chironomus acidophilus* Keyl, 1960 etc.)

**Figure:**
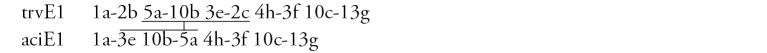


**Arm C** (Fig. 2d, j) has two banding sequences, trvC1 and trvC2, differing by one simple inversion (Fig. 2j). The sequence trvC1 is formed by four inversion steps from a basic sequence, (lonC1), found in several *Chironomus* species (*Chironomus longistylus* Goetghebuer, 1921, *Chironomus anthracinus* Zetterstedt, 1860 etc.).

**Figure 2g-j.**
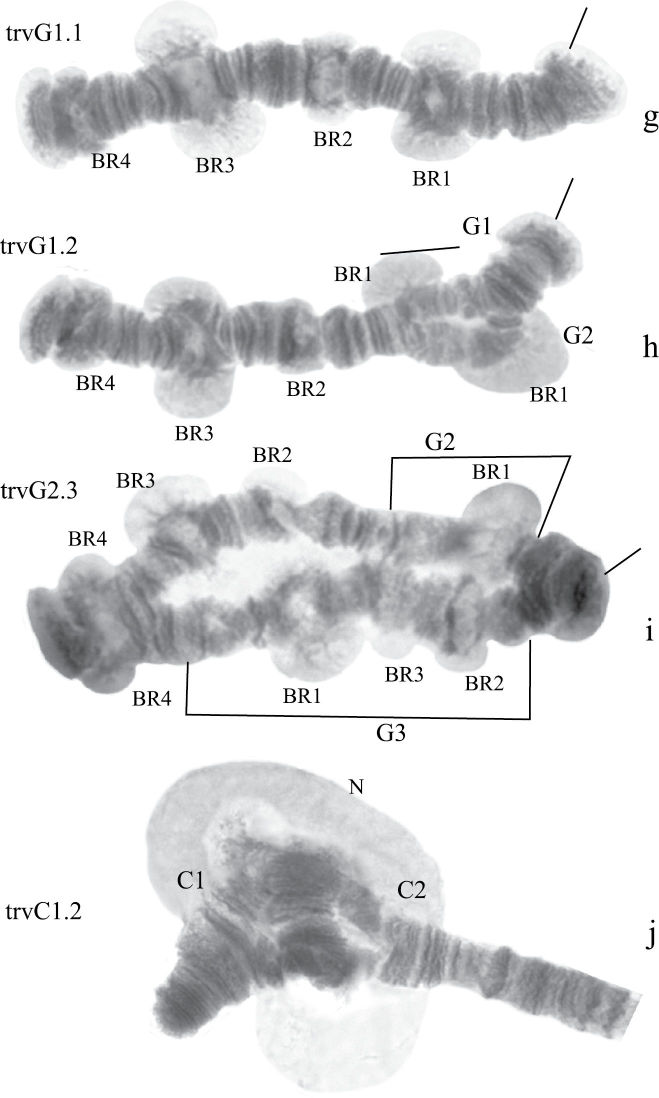
Homozygous and heterozygous banding sequences of *Chironomus transvaalensis* in arm G (**g–i**) and heterozygous inversion in arm C (**j**). Brackets above arms indicate the localization of inversions. The designations are the same as in Fig. 1.

**Figure:**
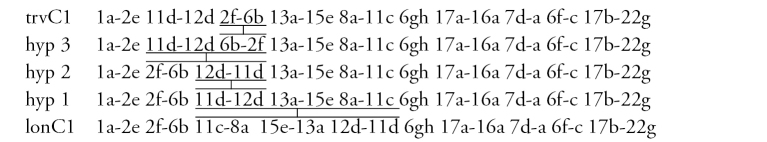


**Arm D** (Fig. 2e) has the sequence trvD1 differing from pigST by four inversion steps.

**Figure:**
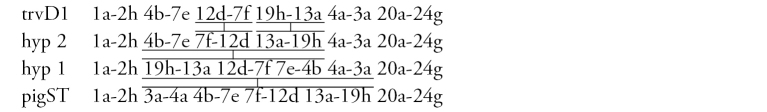


**Arm B** (Fig. 2a) not mapped, monomorphic. BR is well developed.

**Arm F** (Fig. 2f) has the banding sequence trvF1 differing from cosmopolitan basic pigST by three inversion steps.

**Figure:**
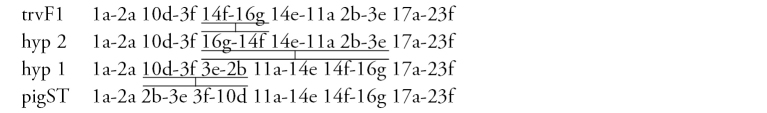


**Arm G** (Fig. 2g-i) has three banding sequences, trvG1, trvG2, and trvG3. The sequence trvG2 differs from trvG1 by a short inversion in the region BR1 (Fig. 2h); the sequence trvG3 – by long inversion of central part of arm G (Fig. 2i). Both last sequences were found as heterozygotes. There are four Balbiani rings.

In total, the banding sequence pool of *Chironomus transvaalensis* contains 10 sequences, all of them are Ethiopian endemic sequences.

#### Larva:

tubuli laterales at abdominal segment VII. Other characters - Mc Lachlan, 1969.

#### Distribution:

various places in Africa, Freeman (1957); Blantyre Malawi (Mc Lachlan), Wülker, 1957: pool east Lake Victoria, Kikuwi-river, Tanzania (J. Grunewald), Pretoria South Africa, Israel (Martin, personal communication).

### Chironomus sp.

Nakuru

#### Previous report:

Wülker, 1980, banding pattern of arms A, E, and F. This species was not identified as well as *Chironomus* sp. Kisumu because there was no additional possibility to collect larvae for rearing. However, the study of *Chironomus* sp. Nakuru karyotype was very important for comparative analysis of Ethiopian *Chironomus* banding sequences with *Chironomus* sequences of the other continents.

#### Karyotype

(Fig. 3a). Haploid number n=4, arm combination AE CD BF G (“pseudothummi” cytocomplex), centromeric bands not heterochromatinized, nucleoli on arms F and G, Balbiani rings on arms G, B, and A. Chromosomal polymorphism was not recorded.

**Figure 3a.**
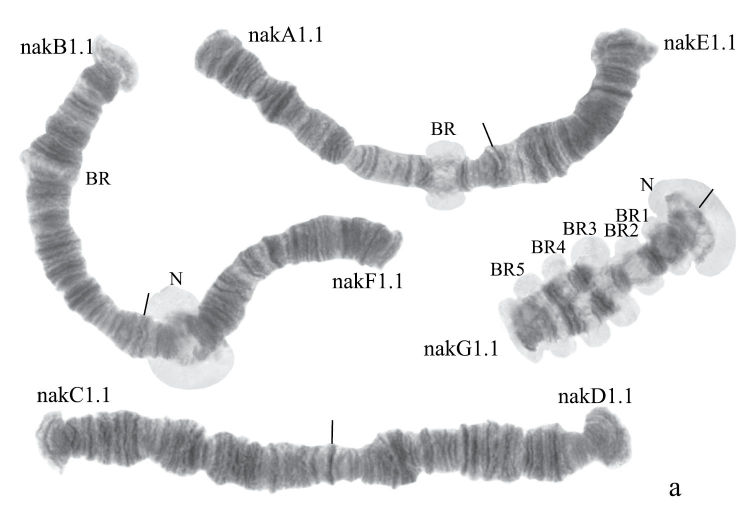
Karyotype of *Chironomus* sp. Nakuru. The designations are the same as in Fig. 2a.

**Figure 3b-f.**
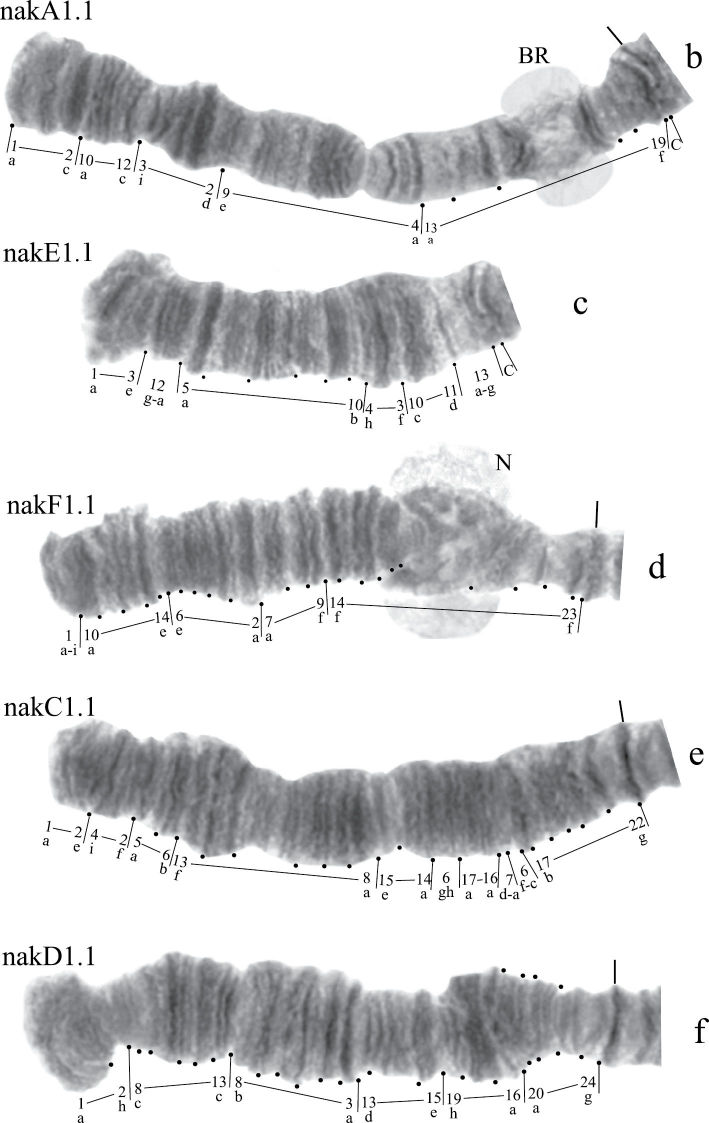
Homozygous banding sequences of *Chironomus* sp. Nakuru in arms A, E, F, C and D. The designations are the same as in Fig. 1.

Banding sequences (Fig 3b-f).

**Arm A** (Fig. 3b) has the banding sequence nakA1 identical with cosmopolitan basic sequence found in many species (*Chironomus holomelas*, *Chironomus melanescens*, etc.)

**Figure:**
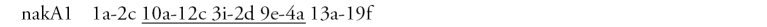


**Arm E** (Fig. 3c) has banding sequence nakE1 differing by two inversion steps from the cosmopolitan basic sequence lonE1 (*Chironomus longistylus*, *Chironomus anthracinus* etc.).

**Figure:**
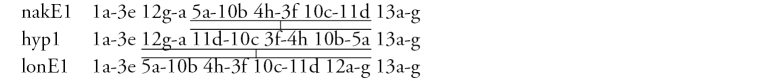


**Arm C** (Fig. 3e) has the sequence nakC1 differing by four inversion steps from basic pattern lonC1 (*Chironomus longistylus*, *Chironomus anthracinus*, etc.) and by seven inversion steps from *Chironomus piger* ST (Fig. 7b).

**Figure 4.**
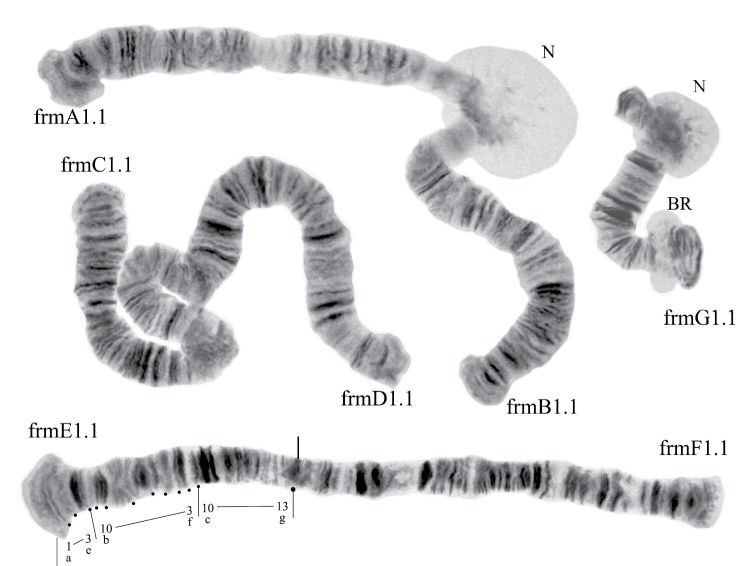
Karyotype of *Chironomus formosipennis*. The designations are the same as in Fig. 1.

**Figure:**
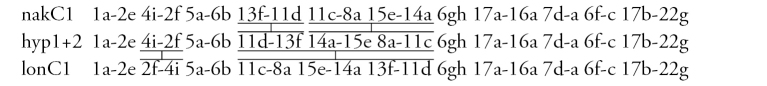


**Arm D** (Fig. 3f) has the banding sequence nakD1 differing from pigST by three inversion steps.

**Figure:**
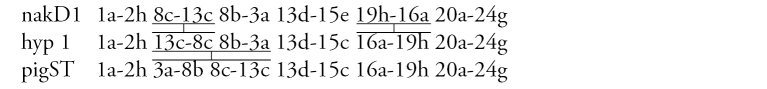


**Arm B** (Fig. 3a) not mapped, monomorphic. The common BR is not developed.

**Arm F** (Fig. 3d) has the banding sequence nakF1 formed by four inversion steps from pigST.

**Figure:**
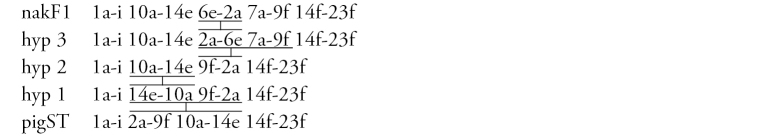


The arm F of *Chironomus* sp. Nakuru has a nucleolus in region 17–19.

**Arm G** (Fig. 3a) has the banding sequence nakG1. It differs from the most of *Chironomus* species arm G by numerous Balbiani rings. It is possible to suggest that some of them can be nucleoli. But it is often impossible to differentiate nucleoli and Balbiani rings without electron microscopy or in situ hybridization.

In total, seven banding sequences are found in sequence pool of *Chironomus* sp. Nakuru, six chromosomal arms have Ethiopian endemic sequences, and one arm (A) a cosmopolitan basic sequence.

#### Larva:

long tubuli laterales at abdominal segment VII, extremely long antenna, gula light, no dark stripe on clypeus.

#### Distribution:

brook to SE of Lake Nakuru, Kenya

### Chironomus formosipennis

Kieffer, 1908

http://species-id.net/wiki/Chironomus_formosipennis

#### Previous reports:

Kieffer 1908, imago.

Freeman 1957, imago.

Dejoux 1970, imago.

Dejoux 1970, pupa.

Dejoux 1970, larva.

#### Karyotype

(Fig. 4). Haploid number n=4, arm combinations AB CD EF G (“thummi” cytocomplex), centromeric bands not heterochromatinized, nucleoli in arms A and G, Balbiani ring in arm G. Chromosomal polymorphism was not recorded.

Banding sequence was determined only in arm E. The sequence frmE1 was identical with the cosmopolitan basic pattern, aprE1 (as in *Chironomus aprilinus* Meigen, 1818)

**Figure:**
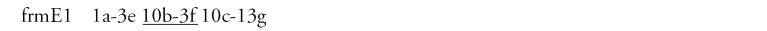


#### Larva:

long tubuli laterales at abdominal segment VII. Other characters - Dejoux 1970.

#### Distribution:

Lake Naivasha, Kenya, Zigi-river, Tanzania, running waters.

### Chironomus prope pulcher

Wiedemann, 1830

#### Previous reports:

Wiedemann 1830, imago.

Freeman 1957, imago.

Dejoux 1968, imago, pupa, larva.

The association to this species is based on one male adult from the collecting sites of the larvae.

#### Karyotype

(Fig. 5a). Haploid number n=3, arm combination AB CD FEG (modified “thummi” cytocomplex), centromeric bands not heterochromatinized, nucleolus in arm F (at the very telomeric end) and nucleolus-like bodies at the ends of arms A, B, E; Balbiani rings are in arms G and B. Chromosomal polymorphism in arm C (Fig. 5a).

**Figure 5a.**
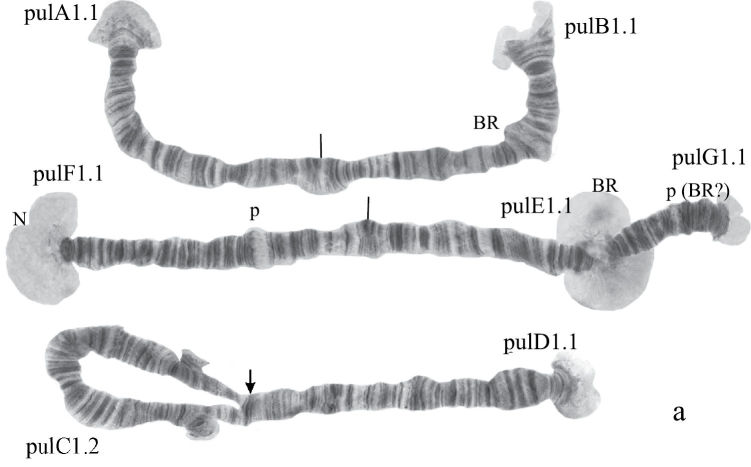
Karyotype of *Chironomus* prope *pulcher.* The designations are the same as in Fig. 1.

**Figure 5b-e.**
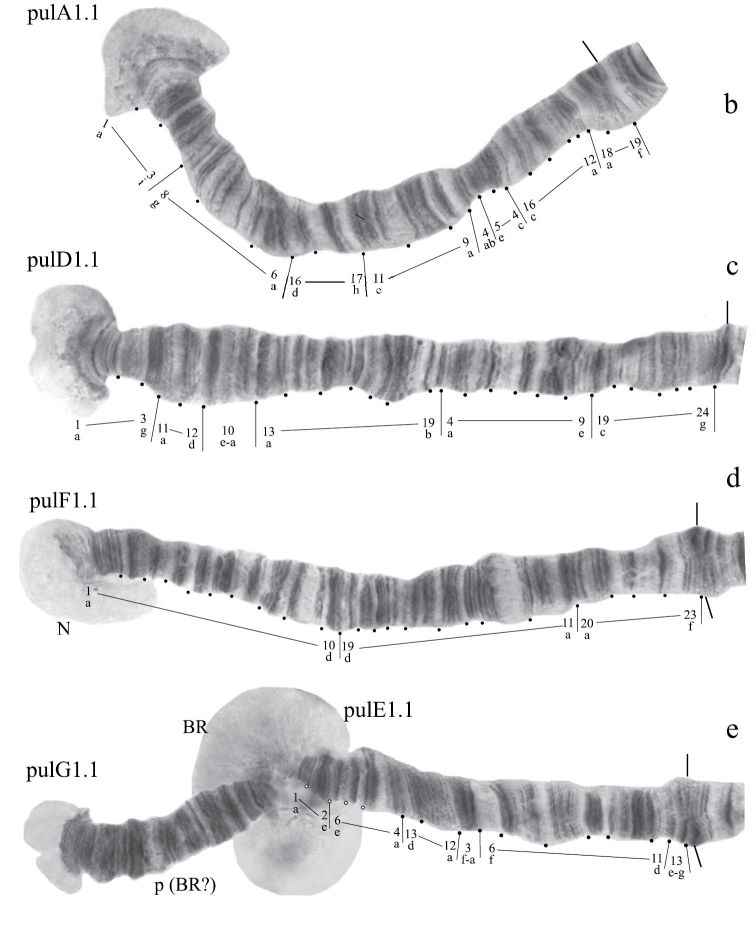
Homozygous banding sequences of *Chironomus prope pulcher* in arms A, D, E, F, and G.

Banding sequences (Fig. 5a, b-e).

**Arm A** (Fig. 5b) has the banding sequence pulA1, formed by four inversions from pigST

**Figure:**
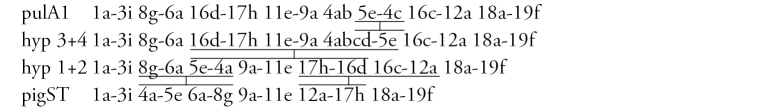


**Arm B** (Fig. 5a) not mapped, monomorphic. It has a sequence pulB1. The common BR is well developed.

**Arm C** (Fig. 5a) not mapped. It has two banding sequences pulC1 and pulC2 differing by a simple inversion, which involved practically the whole central part of arm C.

**Arm D** (Fig. 5c) has the sequence pulD1, formed by five inversion steps from pigST

**Figure:**
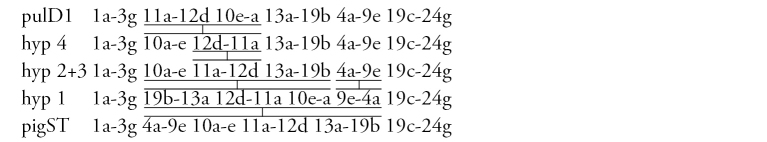


**Arm E** (Fig. 5e) has the banding sequence pulE1, formed by three inversion steps from pigST.

**Figure:**
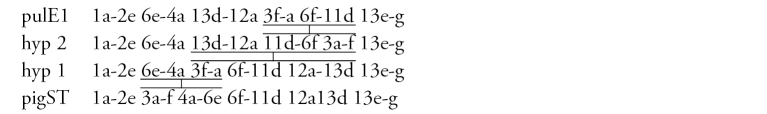


**Arm F** (Fig. 5d) has the sequence pulF1, formed by one simple inversion from pigST.

**Figure:**
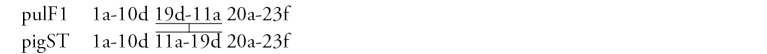


The characteristic of arm F in *Chironomus prope pulcher* is the presence of the nucleolus at the telomeric end, which is a rare event among *Chironomus* species.

**Arm G** (Fig. 5e) is joined with arm E. There is large Balbiani ring near the site of fusion, and a small Balbiani ring or puff in the center of arm G. A small nucleolus is possibly developed at the telomeric end of arm G.

In total, eight banding sequences were recorded in the *Chironomus prope pulcher* banding sequence pool. All of them are endemic for Ethiopia. There are no basic sequences.

#### Larva:

long tubuli laterales on abdominal segment VII. Other characters - Dejoux 1968.

#### Distribution:

two pools within a short distance, River Athi south of Nairobi, Kenya.

### Chironomus sp.

Kisumu

#### Karyotype

**Figure 6b-f.**
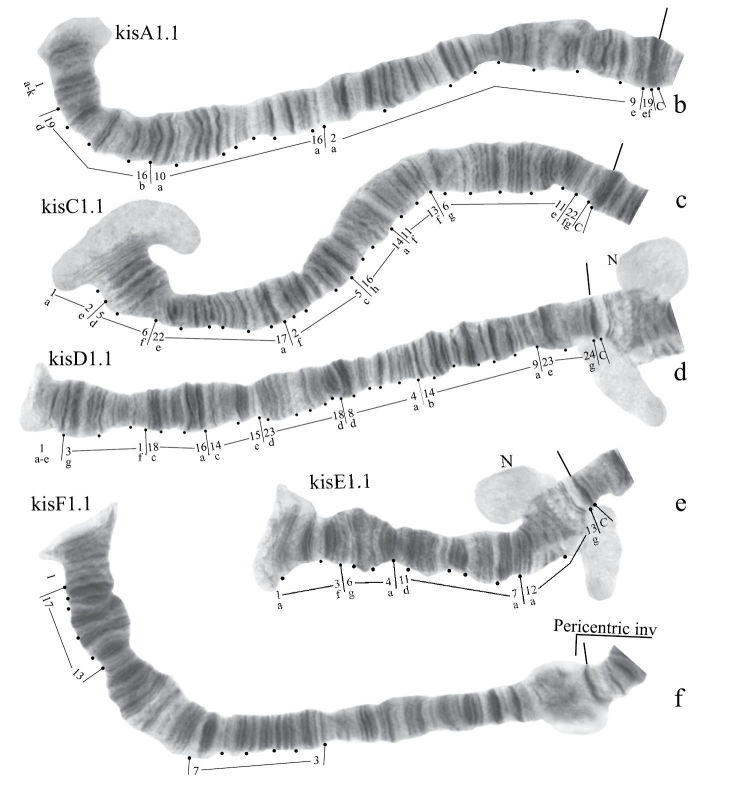
Homozygous banding sequences of *Chironomus* sp. Kisumu in arms A, C. D, E and F.

(Fig. 6a). Haploid number n=4, arm combination AC BF DE G (“parathummi”cytocomplex), centromeric bands not heterochromatinized, nucleoli in arms E and G, Balbiani rings in arms B and G. Chromosomal polymorphism was not recorded.

**Figure:**
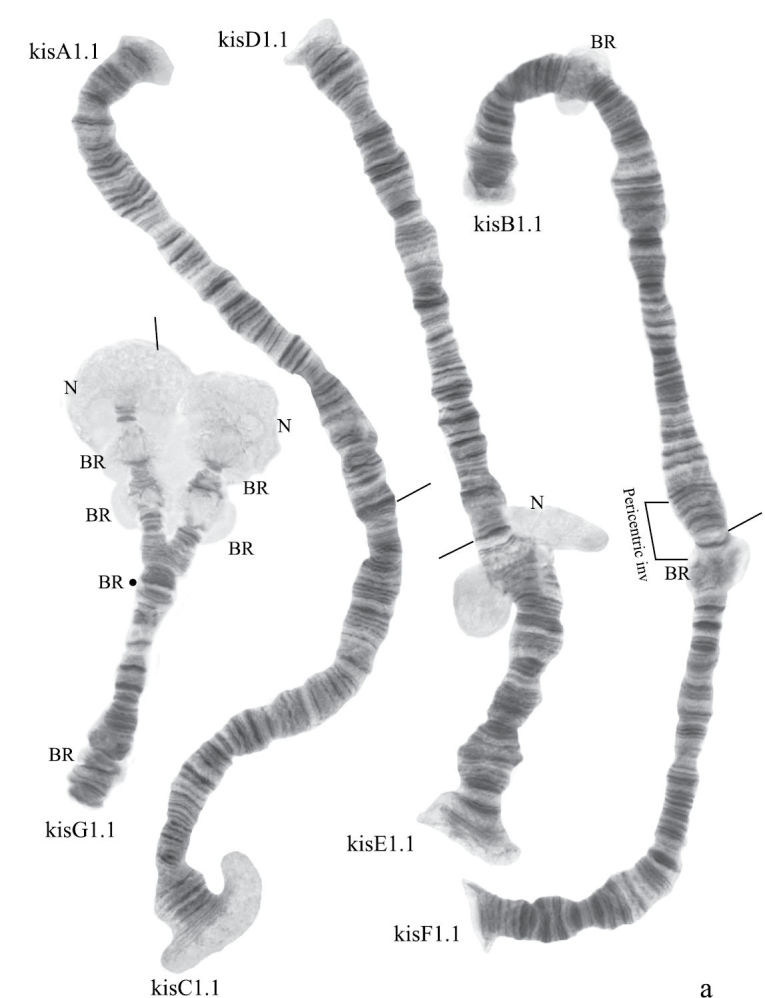
Figure 6a.Karyotype of *Chironomus* sp. Kisumu.

Banding sequences (Fig. 6b-f)

**Arm A** (Fig. 6b) has the sequence kisA1, formed by 3 inversion steps from pigST.

**Figure:**
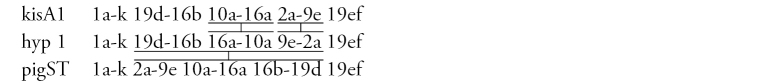


**Arm C** (Fig. 6c) has the sequence kisC1, formed by 8 inversion steps from pigST.

**Figure:**
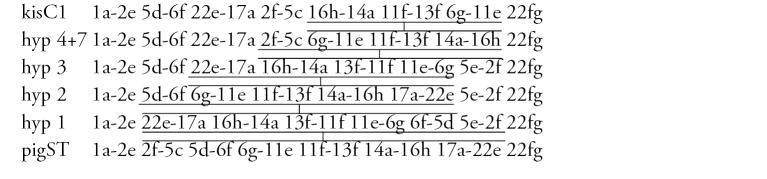


**Arm D** (Fig. 6d) has the sequence kisD1, formed by 6 inversion steps from *Chironomus piger* ST:

**Figure:**
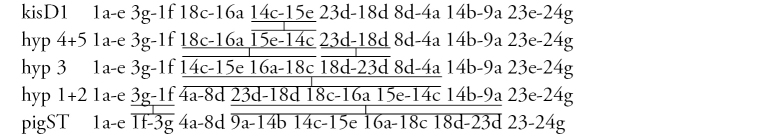


**Arm E** (Fig. 6e) has the sequence kisE1, formed by two inversion steps from *Chironomus piger* ST

**Figure:**
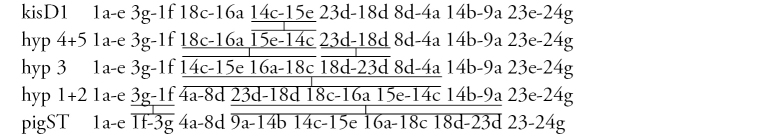


Presence of a nucleolus in region 13 in arm E is a great characteristic of the *Chironomus* sp. Kisumu karyotype.

**Arm B** (Fig. 6a) not mapped. It has one sequence – kisB1. The common BR is well developed.

**Arm F** (Fig. 6f) has the sequence kisF1. It was mapped only fragmentarily because of complex inversions in comparison with *Chironomus piger* ST. The presence of a large Balbiani ring situated just near the centromeric band is a characteristic of arm F in the *Chironomus* sp. Kisumu karyotype. There is pericentric inversion in the chromosome BF (Fig. 6a, f).

**Arm G** (Fig. 6a) is longer than usual in *Chironomus* species. There is a nucleolus and four Balbiani Rings on arm G. One of Balbiani Rings, noted by the black dot in Fig. 6a, was developed only in some cells of the salivary gland cells.

In total, seven Ethiopian endemic banding sequences are found in the sequence pool of *Chironomus* sp. Kisumu. All these sequences differ from *Chironomus parathummi* Keyl, 1961 sequences.

#### Larva:

long tubuli laterales on abdominal segment VII.

#### Distribution:

near Victoria lake, Kenya.

## Discussion

Karyotypes of six African *Chironomus* species were studied. Four of these karyotypes were described for the first time (*Chironomus* sp. Nakuru, *Chironomus formosipennis*, *Chironomus prope pulcher*, *Chironomus* sp. Kisumu). Detailed photomaps of arms A, C, D, E, and F were presented, also for the first time, for *Chironomus alluaudi*, *Chironomus transvaalensis*, and *Chironomus* sp. Nakuru.

Among the species studied, three species (*Chironomus transvaalensis*, *Chironomus prope pulcher*, *Chironomus* sp. Kisumu) have only endemic Ethiopian banding sequences in their karyotypes, while cosmopolitan basic banding sequences were discovered in the karyotypes of the other species, along with endemic sequences (*Chironomus alluaudi*, *Chironomus* sp. Nakuru, *Chironomus formosipennis*). The presence of these basic sequences indicates a relationship of African *Chironomus* species to *Chironomus* species from other continents before their separation (Kiknadze et al. 2008).

The results on African species are relevant the problem whether or not the chromosome arm combination of the “thummi” cytocomplex is rare in Southern continents. At the moment, one species in South America (*Chironomus* sp. Las Brisas, Wülker, Morath, 1989), one species in India (*Chironomus javanus* Kieffer, 1924), and two species in Australia (*Chironomus javanus*, *Chironomus queenslandicus* Martin, 2005) are known to have this “thummi” cytocomplex chromosome arm combination (Martin 2010, Martin, pers. comm. and this paper).

Earlier it was demonstrated (Wülker 1980), that the presence of basic sequences in arms A, E, F of some *Chironomus* species of the “thummi” and “pseudothummi” cytocomplexes supports an idea that the basic sequences existed in hypothetical stem species before the separation of the complexes. The results of this paper contribute to the understanding of chromosome arms C and D in phylogeny in both cytocomplexes, in addition to data on arms A, E and F published earlier (Wülker 1980, 2010, Kiknadze et al. 2008).

**Figure 7a-c.**
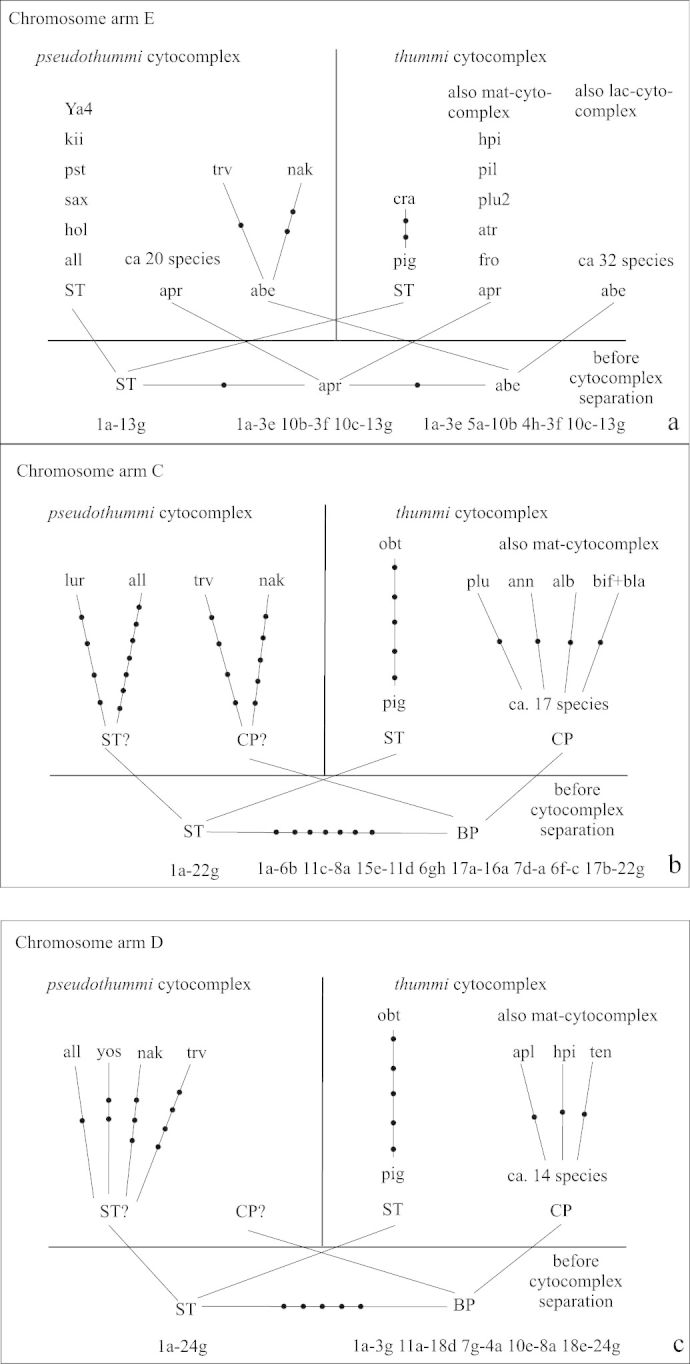
Relations of recent species and hypothetical “basic” species before separation of the cytocomplexes in arm E (**a**), arm C (**b**), and arm D (**c**). The data of Keyl (1962) and Kiknadze (unpublished) were also used. **Dots** – inversion steps between banding sequences; **ST** – piger standard after Keyl (1962) and Dévai et al. (1989). **all** – *alluaudi*, **atr** – *atrella*, **apr** – *aprilinus*, **abe** – *aberratus*, **cra** – *crassicaudatus*, **fro** – *frommeri*, **hol** – *holomelas*, **hpi** – *heteropilicornis*, **kii** – *kiiensis*, **pil** – *pilicornis*, **plu** – *plumosus*, **pst** – *pseudothummi*, **sax** – saxatilis, **trv** – *transvaalensis*.

Keyl (1962) established the hypothesis, that “the hypothetical species, which crossed the border between “thummi” and “pseudothummi” cytocomplexes” had most probably three banding patterns in arm E (in Keyl’s terms): standard as *Chironomus piger* Strenzke, 1959, pattern as *Chironomus aprilinus* Meigen, 1838 and others, pattern as *Chironomus aberratus* Keyl,1961 and others. We can ask, whether these three patterns are known today in both cytocomplexes. This is indeed so (Fig. 7a) with the exception of the fact that the pattern of *Chironomus aberratus* itself is not known in the “pseudothummi” cytocomplex, but there are the sequences trvE1 and nakE1 which differ only by 1–2 inversions from abeE (Fig. 7a).

In arms C and D, an accumulation of species with the identical sequences was previously observed only in the “thummi” cytocomplex (Wülker 2010). With the data of this paper we can propose that chromosome arms C and D had also two patterns before separation of the cytocomplexes (*Chironomus piger* ST sequence sensu Keyl 1962 and basic pattern sensu Wülker 1980, 2010). Fig. 7b shows that pattern ST and basic themselves are not found in “pseudothummi”cytocomplex (question marks in Fig. 7b), but there are several species, which have banding patterns differing only by a few inversions from ST and basic. The African species (*Chironomus alluaudi*, *Chironomus transvaalensis*, *Chironomus* sp. Nakuru, *Chironomus formosipennis*) play an important role in the development of the arm C and D phylogeny. Fig. 7c demonstrates that there are ST and basic patterns in the ‘thummi’cytocomplex, but only patterns close to ST were found in the “pseudothummi”cytocomplex: allD1 only by one, yosD1 by two, nakD1 by three, and trvD1 by four inversions from ST.

A great peculiarity of some African *Chironomus* karyotypes is the presence of large numbers of functionally active chromosome sites, especially Balbiani rings. For example 5 BRs were found in *Chironomus transvaalensis* (Figs 2a, 2g-i), 6 BRs in *Chironomus* sp. Nakuru (Fig. 3a). Most *Chironomus* species have two or three visible BRs since e.g. many species have the gene for BR4 but do not express it, and the number seen may also vary with developmental stage.

## Supplementary Material

XML Treatment for Chironomus alluaudi

XML Treatment for Chironomus transvaalensis

XML Treatment for Chironomus sp.

XML Treatment for Chironomus formosipennis

XML Treatment for Chironomus prope pulcher

XML Treatment for Chironomus sp.
